# The Impact of Intraoperative Frozen Section on Resection Margin Status and Survival of Patients Underwent Pancreatoduodenectomy for Distal Cholangiocarcinoma

**DOI:** 10.3389/fonc.2021.650585

**Published:** 2021-05-03

**Authors:** Zhiqiang Chen, Bingran Yu, Jiaping Bai, Qiong Li, Bowen Xu, Zhaoru Dong, Xuting Zhi, Tao Li

**Affiliations:** ^1^ Department of Hepatobiliary Surgery, General Surgery, Qilu Hospital, Cheeloo College of Medicine, Shandong University, Jinan, China; ^2^ Qilu Hospital, Cheeloo College of Medicine, Shandong University, Jinan, China

**Keywords:** pancreatoduodenectomy, resection margin, survival analysis, frozen section, distal cholangiocarcinoma

## Abstract

**Background:**

Intraoperative frozen section (FS) is broadly used during pancreaticoduodenectomy (PD) to ensure a negative margin status, but its survival benefits on obtaining a secondary R0 resection for distal cholangiocarcinoma (dCCA) is controversial and unclear.

**Methods:**

Clinical data of 107 patients who underwent PD for dCCA was retrospectively collected and divided into different groups based on use of FS (FS and non-FS groups) and status of resection margin (pR0, sR0 and R1 groups), and clinical parameters and survival of patients were compared and analyzed accordingly.

**Results:**

There were 50 patients in FS group with a median survival of 28 months, 57 patients in non-FS group with a median survival of 27 months. There was no statistical difference between the two groups with Kaplan-Meier survival analysis (*P* = 0.347). There were 98 patients in R0 group (88 in pR0 and 10 in sR0) and nine patients in R1 group, with a median survival of 29 months and 22 months respectively, which showed a better survival in R0 group than in R1 group (*P* = 0.006). Survival analyses between subgroups revealed difference between pR0 and R1 group (*P* = 0.005), while no statistical difference concerning pR0 *vs*. sR0 (*P* = 0.211) and sR0 *vs*. R1 groups (*P* = 0.262). Multivariate Cox regression analysis revealed resection margin status, pre-operative biliary drainage and lymph node invasion to be independent prognostic factors for dCCA patients.

**Conclusions:**

Intraoperative FS should be recommended as it significantly increased the rate of R0 resection, which was positively related to a better survival. A primary R0 resection should also be encouraged and if not, a secondary R0 could be considered at the discretion of surgeons as it showed similar survival with primary R0 resection.

## Introduction

Cholangiocarcinoma (CCA), a cancer arising from epithelium of biliary tract, is the most common malignancy in biliary duct system and the second common primary liver malignancy in the whole hepatobiliary system after hepatocellular carcinoma (HCC), accounting for about 3% of all gastrointestinal tumors and 10% to 15% of hepatobiliary malignancies (1, 2). According to the updated 3^rd^ edition of International Classification Diseases for Oncology (ICD-O) system, CCA are categorized into intrahepatic CCA (iCCA), perihilar CCA (pCCA) and distal CCA (dCCA), accounting for about 5% to 10%, 60% to 70%, and 20% to 30% of all CCA cases, respectively (2, 3). Given their differences in frequency, pathobiology, management and prognosis, iCCA, pCCA and dCCA should be viewed as separate entities, and surgery is the only curative treatment for a long-term survival (4). Compared to its two counterparts, dCCA is usually indicated for pancreaticoduodenectomy (PD) or pylorus-preserving pancreaticoduodenectomy (PPPD) with lymphadenectomy which has a higher resectability rate than iCCA or pCCA (5). However, the overall survival of dCCA patients after curative resection was still dismal, with a 5-year survival rate about 18% to 43% (5–7).

Resection margin status is considered to be a major prognostic factor of survival for dCCA patients, thus a R0 resection is always pursued by surgeons (7–9). And the only method of assessing intraoperative resection margin status was frozen section (FS) (10, 11), but the clinical value of FS on assessing bile duct resection margin is controversial and debated because of its inherent pitfalls and probable disagreement with permanent section (PS) (12, 13). In this study we retrospectively analyzed survival outcomes in patients with dCCA who underwent PD for curative resection, aiming to investigate if the use of intraoperative FS could provide survival benefits by obtaining a secondary R0 resection margin. Furthermore, independent prognostic factors of dCCA were also investigated via multivariable Cox regression analysis.

## Methods

### Study Design and Participants

A retrospective analysis was conducted on patients who underwent PD for dCCA in the General Surgery Department of Qilu Hospital (Cheeloo College of Medicine, Shandong University) from January 2011 to November 2019. dCCA was defined as carcinoma arising from distal part of extrahepatic bile duct that was below insertion of cystic duct. Only patients with pathologically confirmed dCCA that underwent curative PD were included; patients undergoing PD for diseases other than dCCA (such as pancreatic cancer, duodenal cancer, ampullary cancer and benign lesions) were excluded.

All relative clinical data were collected including baseline demographics, tumor characteristics, as well as long-term follow-up for patients survival. Primary analysis was performed between FS and non-FS groups in order to view if the use of FS had some impact on patient overall survival. Then, a further statistical analysis comparing different margin status were performed to see if the use of FS on obtaining a sR0 could improve long-term survival of patients. Thus, patients were divided into pR0, sR0 and R1 groups accordingly, and clinical parameters and survival of patients were compared and analyzed among groups.

R0 was defined as absence of macroscopic and microscopic tumor cells at the bile duct margin, with two subgroups of pR0 (primary R0) and sR0 (secondary R0, a negative margin achieved by extended resection). R1 was defined as presence of microscopic tumor cells at resection margin, while R2 resection was defined as macroscopically visualized tumor at margins. Clavien-Dindo classification was used to analyze the postoperative complication of patients (14). Tumor staging was classified according to the 8th edition of TNM classification system from American Joint Committee on Cancer (AJCC) (15).

### Statistical Analysis

The numerical data were expressed as mean ± standard deviation and assessed by using the Student’s *t*-test. The categorical data were presented as percentage and assessed by the chi-square test. Analysis of variance (ANOVA) was used to compare the data among three groups. Postoperative survival was described by Kaplan–Meier curves and comparison between groups was performed using log-rank test. The Cox proportional hazard model was used for multivariable analysis. A *P* value of less than 0.05 was considered statistically significant. Analyses were performed with SPSS 25.0 (SPSS, Chicago, IL, USA).

## Results

### Patient Demographics

**Figure 1:**
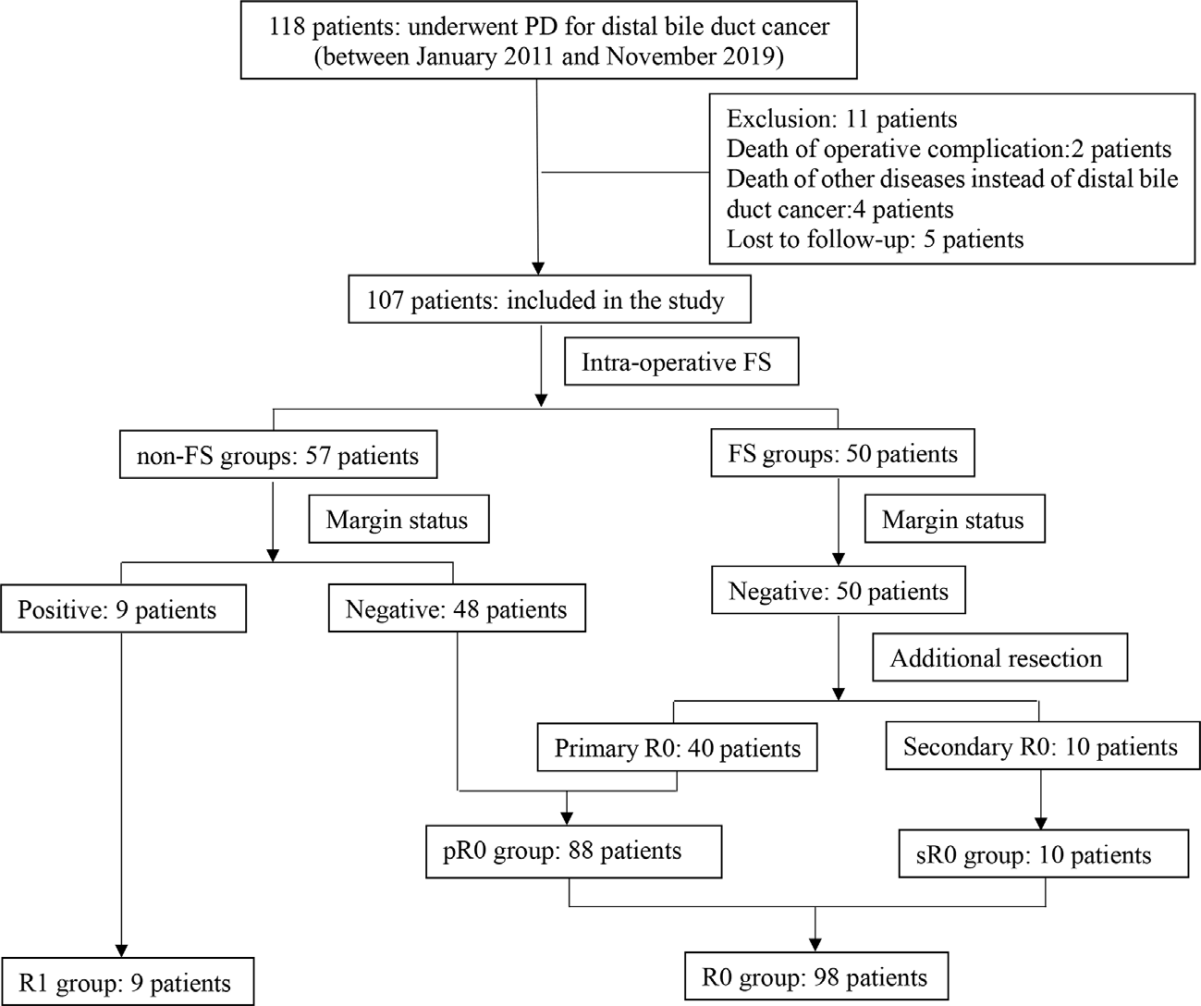
Flow chart of patient selection and classification of the study. 107 patients were included and divided into different groups based on use of FS (FS and non-FS groups) and resection margin status (pR0, sR0 and R1 groups).

The patient selection and demographics were summarized and shown in **Figure 1** and **Table 1**. Briefly, there were 107 patients with a mean age of 62.3 years and a male to female ratio of 70:37. There were 50 patients in FS group and 57 patients in non-FS group; while there were 98 patients in R0 group (88 pR0 plus 10 sR0) and nine patients in R1 group. Preoperative biliary drainage was performed in 71 patients via percutaneous transhepatic cholangial drainage (PTCD, 64 cases), endoscopic nasobiliary drainage (ENBD, three cases) and endoscopic retrograde biliary stent (ERBS, four cases), respectively. Only 33 (30.8%) patients received post-operative chemotherapy and/or radiotherapy.

**Table 1:** Patient demographics and clinicopathologic characteristics.

	Total (N = 107)	Subgroups by FS			Subgroups by resection margin status			
		non-FS group (n = 57)	FS group (n = 50)	*P*-value	pR0 group (n = 88)	sR0 group (n = 10)	R1 group (n = 9)	*P*-value
Age (mean ± SD, years)	62.3 ± 8.21	62.0 ± 8.46	62.7 ± 7.96	0.637	62.2 ± 8.14	60.5 ± 9.55	65.6 ± 7.32	0.388
Sex				0.486				0.106
Male	70 (65.4%)	39 (68.4%)	31 (62.0%)		60 (68.2%)	7 (70.0%)	3 (33.3%)	
Female	37 (34.6%)	18 (31.6%)	19 (38.0%)		28 (31.8%)	3 (30.0%)	6 (66.7%)	
Complication	40 (37.4%)	23 (40.4%)	17 (34.0%)	0.498	33 (37.5%)	4 (40.0%)	3 (33.3%)	0.955
CA199 (mean, U/ml)	242.1 ± 297.44	255.0 ± 317.86	227.3 ± 274.78	0.634	233.9 ± 295.41	167.8 ± 150.86	404.0 ± 400.64	0.188
Pre-operative biliary drainage	64 (90.1%)	37 (64.9%)	34 (68.0%)	0.736	58 (65.9%)	9 (90.0%)	4 (44.4%)	0.108
Pancreatoduodenectomy	100 (93.5%)	53 (93.0%)	47 (94.0%)	0.832	83 (94.3%)	9 (90.0%)	8 (88.9%)	0.737
Clavien-Dindo classification				0.841				0.522
I	61 (57.0%)	31 (54.4%)	30 (60.0%)		51 (58.0%)	5 (50.0%)	5 (55.6%)	
II	14 (13.1%)	8 (14.0%)	6 (12.0%)		13 (14.8%)	0 (0%)	1 (11.1%)	
III	32 (29.9%)	18 (31.6%)	14 (28.0%)		24 (27.3%)	5 (50.0%)	3 (33.3%)	
Tumor differentiation				0.299				0.723
Low	46 (43.0%)	22 (38.6%)	24 (48.0%)		39 (44.3%)	4 (40.0%)	3 (33.3%)	
Moderate	51 (47.7%)	31 (54.4%)	20 (40.0%)		42 (47.7%)	5 (50.0%)	4 (44.4%)	
High	10 (9.3%)	4 (7.0%)	6 (12.0%)		7 (8.0%)	1 (10.0%)	2 (22.2%)	
Tumor size (mean, cm)	2.0±0.66	1.9±0.81	2.0±0.82	0.846	1.9±0.76	2.4±1.23	2.1±0.67	0.182
T stage				0.808				0.985
T1/2	33 (30.8%)	17 (29.8%)	16 (32.0%)		27 (30.7%)	3 (30.0%)	3 (33.3%)	
T3	74 (69.2%)	40 (70.2%)	34 (68.0%)		61 (69.3%)	7 (70.0%)	6 (66.7%)	
Pancreatic invasion	67 (62.6%)	37 (64.9%)	30 (60.0%)	0.600	54 (61.4%)	6 (60.0%)	7 (77.8%)	0.615
Duodenal invasion	17 (15.9%)	12 (21.1%)	5 (10.0%)	0.119	15 (17.0%)	1 (10.0%)	1 (11.1%)	0.778
Microvascular invasion	20 (18.7%)	11 (19.3%)	9 (18.0%)	0.864	18 (20.5%)	1 (10.0%)	1 (11.1%)	0.601
Lymph invasion	24 (22.4%)	12 (21.1%)	12 (24.0%)	0.715	21 (23.9%)	1 (10.0%)	2 (22.2%)	0.609
Perineural invasion	36 (33.6%)	19 (33.3%)	17 (34.0%)	0.942	29 (33.0%)	5 (50.0%)	2 (22.2%)	0.418
Adjuvant treatment	33 (30.8%)	19 (33.3%)	14 (28.0%)	0.551	23 (26.1%)	5 (50.0%)	5 (55.6%)	0.074

### Clinical Outcomes Between FS Group and Non-FS Group

There were 50 patients in FS group and 57 patients in non-FS group. As shown in **Table 1**, there was no statistical difference between the two groups concerning baseline demographics and tumor characteristics (*P*>0.05). There was no false negative report for FS in our study, as all the R0 resection margin in FS group was confirmed by postoperative PS. While nine patients in non-FS group were revealed to be R1 resection that was confirmed by postoperative PS, making the rate of R1 resection in non-FS groups higher than that of FS group (15.8% *vs*. 0%, *P* = 0.003).

**Figure 2:**
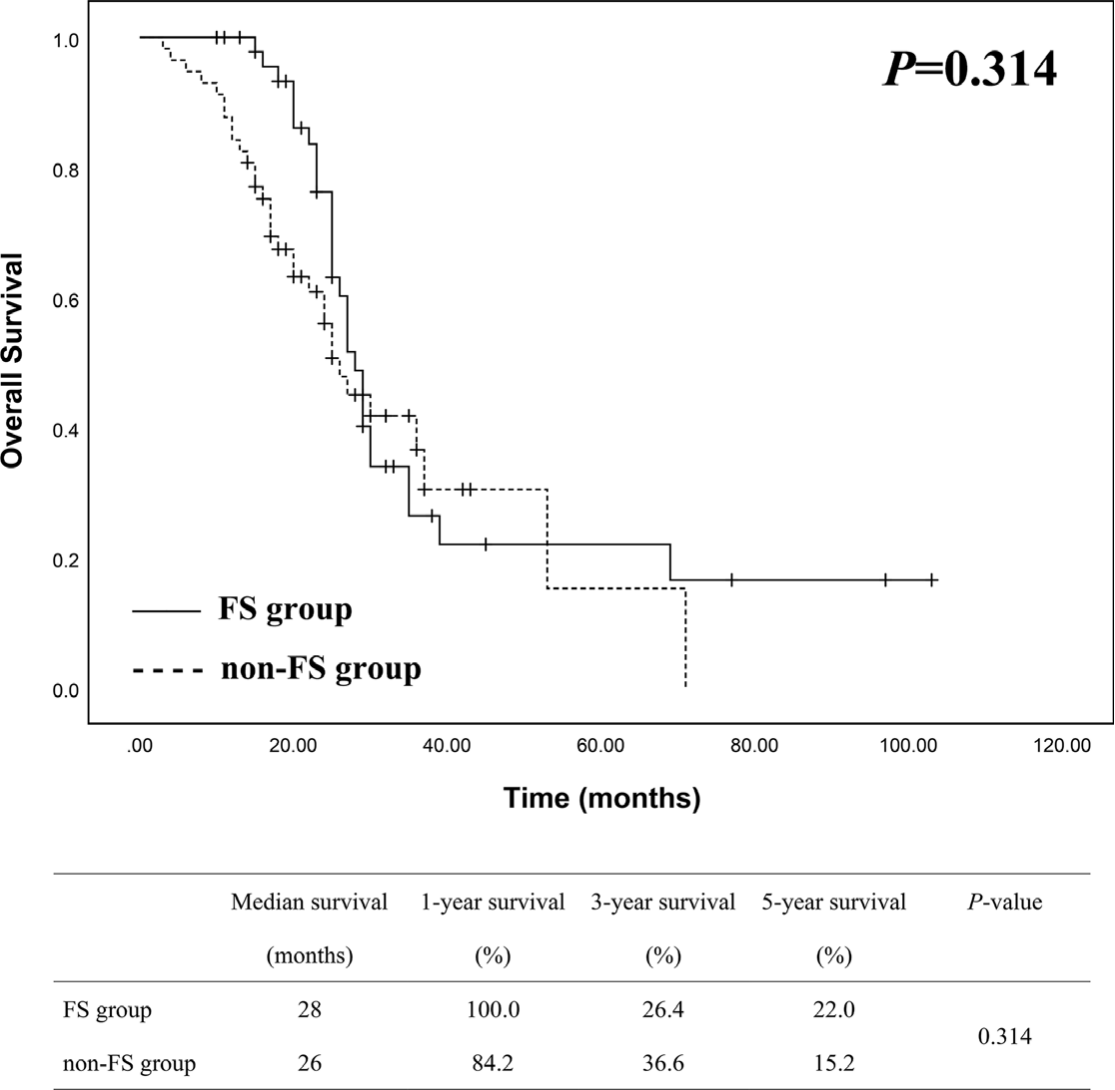
Survival analysis between FS (n = 50) and non-FS (n = 57) groups showed no statistical difference.

Patient survival of the two groups was demonstrated in **Figure 2**. The median survival was 28 months in FS group and 26 months in non-FS group, while the 1-, 3- and 5-year survival of FS group was 100%, 26.4% and 22.0%, compared to 84.2%, 36.6% and 15.2% in non-FS group, respectively. There was no statistical difference of overall survival between the two groups by Kaplan-Meier analysis (*P* = 0.314).

### Influences of Resection Margin Status on Clinical Outcomes

**Figure 3:**
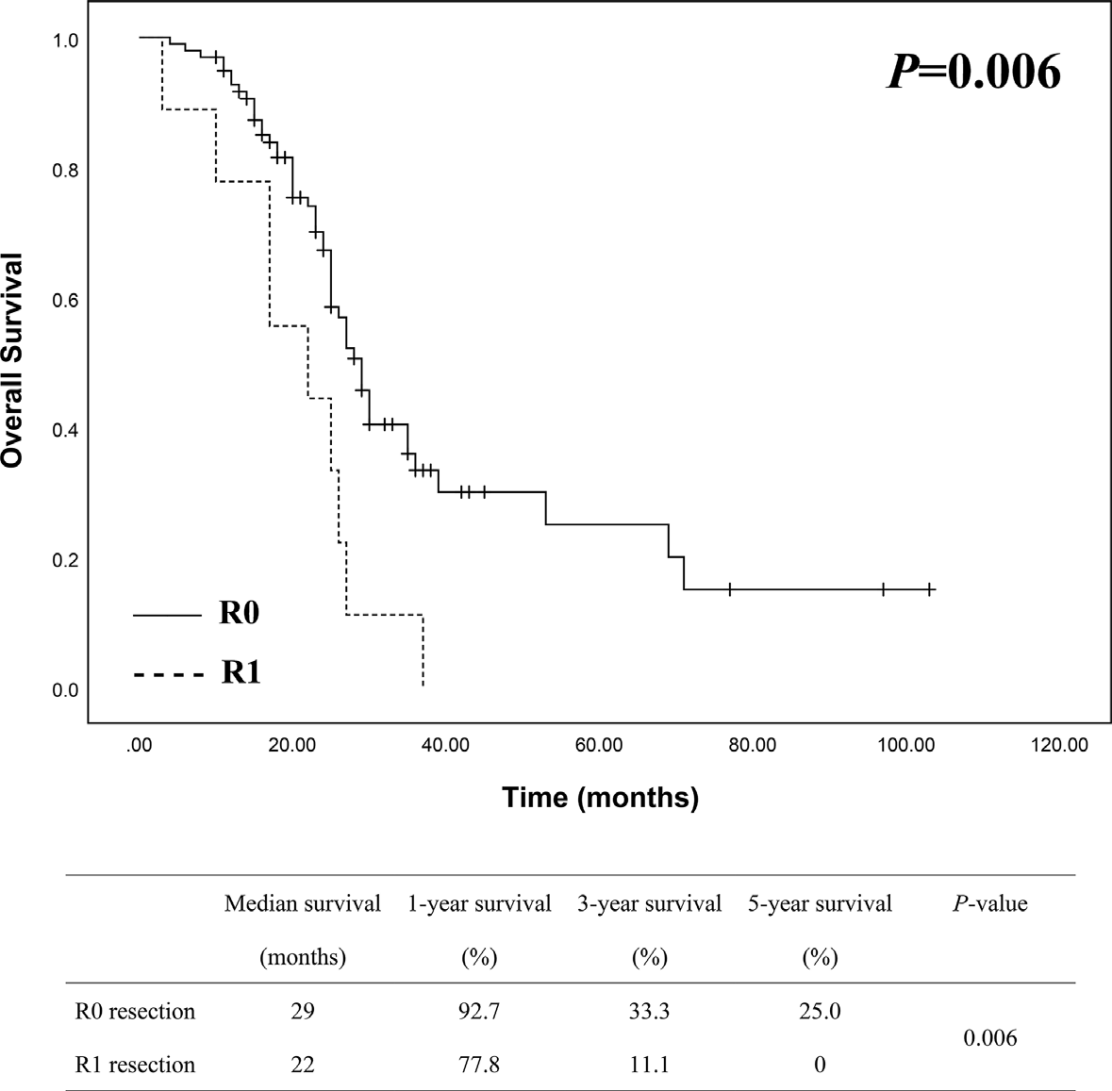
Survival analysis between R0 (n = 98) and R1 (n = 9) groups showed a better survival of R0 resection with a statistically significant difference.

In order to view if survival of patients may differ between resection margin status, we divided the patients into two groups, namely R0 group (98 patients) and R1 group (9 patients). Kaplan-Meier analysis was then used to compare survival differences between the two groups. As shown in **Figure 3**, the median survival was 29 months in R0 group and 22 months in R1 group, while the 1-, 3- and 5-year survival in R0 group was 92.7%, 33.3% and 25.0% compared to 77.8%, 11.1% and 0 in R1 group, which showed a better survival in R0 group of patients than in R1 group with a statistically significant difference (*P* = 0.006).

**Figure 4:**
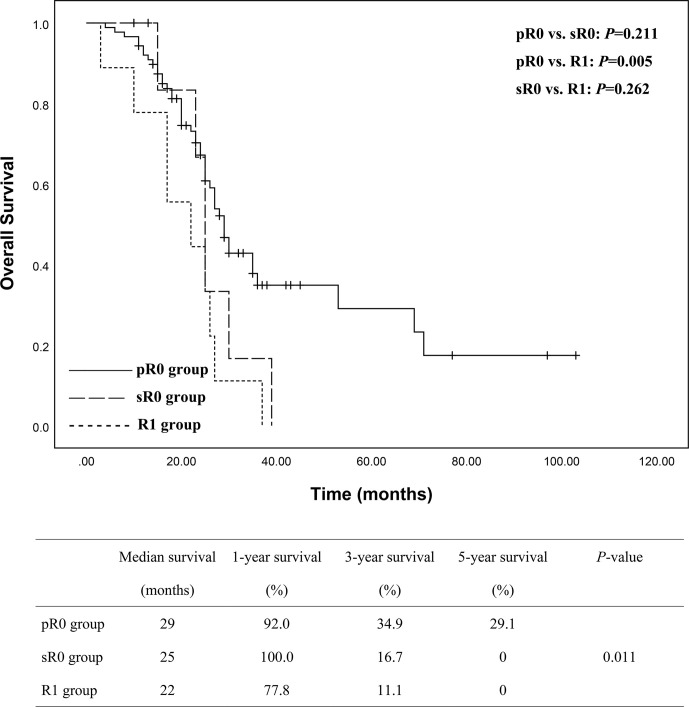
Subgroups analyses of the overall survival between pR0 (n = 88), sR0 (n = 10) and R1 (n = 9) groups showed a significant better survival for pR0 group of patients, but no statistical differences between sR0 *vs*. pR0 and sR0 *vs*. R1.

To assess the impact of additional resection to achieve a secondary R0 resection margin, we subdivided R0 group into pR0 (88 patients) and sR0 (10 patients) as mentioned above. Statistical analysis among pR0, sR0 and R1 groups was conducted with Kaplan-Meier curve and illustrated in **Figure 4**, which showed significant difference in terms of survival among the three groups (*P* = 0.011). A more detailed analysis between subgroups was then conducted, revealing that there was significant difference between pR0 and R1 group (*P* = 0.005), while no statistical difference was observed concerning pR0 *vs*. sR0 (*P* = 0.211) and sR0 *vs*. R1 groups (*P* = 0.262).

### Multivariable Cox Regression Analysis for Risk Factors on Overall Survival

On multivariate Cox regression analysis, independent factors associated with reduced OS were revealed to be pre-operative biliary drainage, positive lymph invasion and R1 resection margin (**Table 2**). It was notable that conversion of R1 to R0 (i.e. sR0) presented a similar survival with pR0 group, with a HR of 1.089 (95% CI = 0.438–2.705, *P* = 0.854), while R1 resection showed a poor survival with a HR of 3.233 (95% CI = 1.517–6.890, *P* = 0.002).

**Table 2:** Multivariate analysis of variables associated with overall survival.

Variable	Median survival (months)	5-year survival (%)	Hazard ratio	95% CI	*P*-value
Sex					0.066
Male	28	38.0	1.00 (reference)		
Female	26	7.9	1.70	0.965–2.985	
Biliary drainage					0.033
No	29	37.4	1.00 (reference)		
Yes	25	13.2	1.94	1.056–3.580	
Clavien-Dindo classification					0.101
I	29	24.3	1.00 (reference)		
II	53	25.5	0.69	0.265–1.794	
III	25	18.2	1.63	0.917–2.889	
Lymph invasion					0.017
Negative	29	26.2	1.00 (reference)		
Positive	20	0	2.21	1.151–4.255	
Margin status					0.010
pR0	29	29.1	1.00 (reference)		
sR0	25	0	1.089	0.438–2.705	0.854
R1	22	0	3.233	1.517–6.890	0.002

## Discussion

Although the incidence rate of dCCA is decreasing and resectability is increasing with medical developments over last decades, the long-term survival of patients is still dismal (5, 6). In this study, there were 107 patients who underwent PD or PPPD for dCCA in our hospital during the study period, with a median survival of 27 months, 1-, 3- and 5-year survival of 91.5%, 31.3% and 21.5%, respectively, which were in consistence with other reports (7, 16).

Surgery still remains the only potentially curative treatment for dCCA patients, and intraoperative FS is broadly used to ensure a R0 resection margin which is considered to be an important prognostic factor for long-term survival (7–9). However, the use and clinical value of FS is controversial as some pitfalls and disagreement with permanent section (PS) may present. For example, the diagnosis of some lesions (severe dysplasia, carcinoma *in situ*, and intraepithelial neoplasia) might be subjective due to the lack of standard diagnostic criteria (17, 18). Preoperative manipulation, such as biliary drainage, stenting or biopsy, might lead to inflammation, fibrosis and other reactive changes of epithelium which may influence the diagnosis of resection margin (11, 17–19). Besides, it was challenging even for experienced pathologists to differentiate true cytologic atypia and reactive gland from invasive carcinoma (19, 20). There were several studies focusing on the use of FS during PD procedure for pancreatic cancer, that showed FS did improve the rate of R0 resection by 6.0% to 8.4%, but with no evidence of improved survival by extending the pancreatic resection to obtain a sR0 resection margin (21–24). But limited data is known about the use of FS to obtain a sR0 resection margin and its impact on survival of dCCA patients.

In present study, we demonstrated that intraoperative use of FS significantly increased the rate of R0 resection, but it did not improve the overall survival of patients. Nevertheless, our further analysis revealed that R0 resection had a significant better survival than R1 resection. Indeed, most studies agreed that R1 resection had a strong correlation with local recurrence, and complete R0 resection was a major prognostic factor for long-term survival of CCA patients (7–9). But there were some other reports showed that patients underwent R1 resection lived longer than expected, and it seemed that most of them had a positive ductal margin of carcinoma *in situ* (not invasive carcinoma) (25, 26). It needs to be mentioned that in our study, there was one patient in FS group having carcinoma *in situ* at the primary resection margin and was converted to sR0 by additional resection; and there was another patient in non-FS group having carcinoma *in situ* at the resection margin revealed by postoperative PS which was allocated to R1 group. Both patients underwent postoperative chemotherapy and survived till last follow-up (21 and 29 months). Long-term follow-up for this kind of patients should be emphasized, as they may have better survival than invasive carcinoma at resection margin.

Next, we subdivided the R0 group into pR0 and sR0 to assess if the use of FS on ensuring a sR0 could provide some survival benefits. Data revealed that pR0 group of patients shared the best long-term survival than R1 group, while sR0 group did not show statistical difference compared to R1 group. But it is of interest that survival of sR0 group and pR0 group did not show statistical difference either, which mean they may have similar survival. Here, we can see a statistical dilemma between these three groups (i.e. pR0 *vs*. R1, sR0 *vs*. R1 and sR0 *vs*. pR0), we think it is related to two reasons: firstly, the follow-up time was not long enough for all patients, and there were still some survivors at the time of last follow-up; secondly, the numbers of patients in sR0 and R1 group were small which could affect the power of statistical analyses. Thus, long-term follow-up and large number of patients should be emphasized for future studies.

Nevertheless, the present data showed that R0 resection provided a significantly better survival than R1 resection, especially for pR0 resection group. Intraoperative FS should be recommended as it could dramatically increase R0 resection rate during operation, and re-resection of positive margin should be considered at the discretion of experienced surgeons as sR0 resection showed similar survival with pR0 resection in our study. Indeed, many studies approved additional resection to achieve sR0 resection for pCCA and eCCA (extrahepatic cholangiocarcinoma) when the primary resection margin was positive on intraoperative FS (7–9, 26–28). In a recent research, Park et al. compared overall survival between R0 on first bile duct resection (pR0) and R0 after additional resection (sR0), and supposed there was no difference in OS between the two groups (29). In a study by Tsukahara et al., they demonstrated the survival rate of seven patients achieved R0 status after additional resection (sR0) was similar to that of pR0, and suggested additional resection was beneficial to some selected patients with positive margin of carcinoma *in situ* (CIS) (30).

There were many series investigating prognostic factors of surgically treated dCCA patients, and the commonly identified factors were lymph node invasion, pancreatic invasion, perineural invasion, resection margin status, tumor grade, blood transfusion and adjuvant therapy (8, 10, 13, 29, 31–33). Our study revealed that, besides positive resection margin, pre-operative biliary drainage and lymph node invasion were both independent risk factors of poor survival of dCCA patients with surgical resection (**Table 2**). To be noted, pancreatic invasion, perineural invasion, tumor grade (size, differentiation and T stage), blood transfusion and adjuvant therapy were not associated with prognosis in our study groups. Majority of the patients received preoperative biliary drainage to alleviate jaundice, and most of them were treated with PTCD which is more favorable in our hospital. Cox regression model revealed preoperative biliary drainage to be a risk factor for poor survival, as may be explained that patients who received biliary drainage always had more severe obstructive jaundice and comorbidities (a total bilirubin >200 µmol/L and/or severe comorbidities that need to take long time to be improved). A recent investigation by Miura et al. evaluated prognostic impact of the type of preoperative biliary drainage, and concluded that PTCD should be avoided since patients in their cohort who underwent PTCD had poorer overall survival and higher incidence of liver metastasis than those who underwent endoscopic biliary drainage (ENBD+ERBS) (34). As case numbers of ENBD and ERBS in our study were limited, it was inefficient to analyze survival data among subgroups. Future large volume or multicenter randomized control trials should be designed to get this issue clarified. Lymph node invasion was indicated as another independent risk factor for poor survival in our study. Although it has already reached a consensus that lymph node metastasis is a prognostic indicator by most studies (8, 10, 29), whether to take extended lymphadenectomy is still under controversy, as some studies suggested it could not improve survival but help to stage and predict prognosis, while some other studies advocated extended lymphadenectomy for a survival advantage (35–38). Again, randomized control trials should be emphasized for future studies on this issue.

There were some limitations for this study. Firstly, this was a primary study with small sample size, especially for sR0 and R1 groups of patients, and the median follow-up period was 24.0 ± 12.27 months, so the study was subject to an underpowered statistical difference. Secondly, this was a retrospective analysis with some inherent pitfalls such as participant selection bias. Thirdly, this was a single center study and therefore may not be generalizable more broadly. So large volume or multi-center randomized control trials should be designed for future investigation.

To be concluded, although intraoperative FS did not show an overall survival benefit compared to non-FS group in our study, it still should be recommended as it dramatically increased the rate of R0 resection, and R0 resection showed a better survival outcomes compared to R1 group. A pR0 resection should also be encouraged and if not, a sR0 should be considered at the discretion of surgeons as it showed similar survival with pR0 resection. Besides resection margin status, preoperative biliary drainage and lymph node metastasis were also revealed to be independent prognostic factors by multivariate analyses. And multi-center randomized control trials with large patient volume should be considered for future studies.

## Data Availability Statement

The raw data supporting the conclusions of this article will be made available by the authors, without undue reservation.

## Ethics Statement

The studies involving human participants were reviewed and approved by Medical Ethics Committee of Qilu Hospital, Shandong University. The patients/participants provided their written informed consent to participate in this study.

## Author Contributions

Study concepts: ZC, BY, and TL. Study design: ZC, BY, and TL. Data acquisition: BY and JB. Quality control of data and algorithms: ZC, BY, and QL. Data analysis and interpretation: ZC, BY, and ZD. Statistical analysis: ZC, BY, and BX. Manuscript preparation: ZC and BY. Manuscript editing: ZC and BY. Manuscript review: ZC, XZ, and TL. All authors contributed to the article and approved the submitted version.

## Funding

This study was supported by the Taishan Scholars Program for Young Experts of Shandong Province (tsqn20161064) and the National Natural Science Foundation of China (82073200 & 81874178) for TL.

## Conflict of Interest

The authors declare that the research was conducted in the absence of any commercial or financial relationships that could be construed as a potential conflict of interest.
